# Polyethylene liner dissociation from humeral tray: impediment to closed reduction of dislocated reverse total shoulder replacement

**DOI:** 10.1016/j.jseint.2022.10.010

**Published:** 2022-11-09

**Authors:** Michael Doran, Michael A. Boin, Utkarsh Anil, Sebastian Bustamante, Young W. Kwon, Joseph D. Zuckerman, Mandeep S. Virk

**Affiliations:** Division of Shoulder and Elbow, Department of Orthopedic Surgery, New York University New York, NY, USA

**Keywords:** Polyethylene dissociation, Revision TSA, RTSA instability, Closed reduction, Reverse total shoulder arthroplasty

## Abstract

**Background:**

Instability is one of the leading causes of revision for reverse total shoulder arthroplasty (RTSA). Closed reduction (CR) of a dislocated RTSA is recommended by many as initial treatment with varying degrees of success. In this study, we describe polyethylene liner dissociation from the humeral tray (PDH) as a cause of failure of CR of dislocated RTSA.

**Methods:**

In this retrospective study, patients who underwent revision RTSA for instability were identified through our institutional database review using specific International Classification of Diseases and Current Procedural Terminology codes. Pertinent clinical information including demographics, details of instability event (early vs. late), traumatic vs. atraumatic, outcomes of CR (if performed), and intraoperative findings during revision surgery were collected and analyzed.

**Results:**

Twenty-two patients met the inclusion criteria with average follow-up of 2 years. CR was attempted in 12 (55%) patients, prior to revision surgery, and was successful in 5 (23%) patients. During the revision surgery polyethylene liner dissociation from the humeral tray (PDH) was identified in 10 patients (45%). Five of these 10 patients had failed CR and the other 5 patients did not undergo CR due to primary surgeon’s preference. All patients with PDH event had onlay humeral tray RTSA system. Although not a consistent radiographic finding in our series, the presence of the metallic glenosphere in direct contact with the humeral tray on anteroposterior or axillary radiographs was diagnostic for PDH.

**Conclusion:**

Dissociation of polyethylene liner from the humeral tray can be associated with an RTSA dislocation and is a contraindication for CR. A radiographic finding of the metallic humeral tray articulating directly with the glenosphere is an indication that the polyethylene liner is dissociated from the humeral tray.

The number of reverse total shoulder arthroplasties (RTSAs) performed in the United States continues to increase. It has emerged as a successful treatment option for a variety of shoulder pathologies. Good to excellent clinical outcomes have been reported in patients with irreparable rotator cuff tears, proximal humerus fractures, glenohumeral osteoarthritis, and for patients undergoing revision total shoulder arthroplasty.^1^, ^2^, ^3^^,^^8^, ^9^, ^10^, ^11^, ^12^^,^^14^, ^15^, ^16^^,^^18^^,^^19^ Despite recent improvements in implant design and surgical techniques, complications after RTSA remain a considerable concern.^12^^,^^22^

Instability continues to be one of the most frequent complications after RTSA and a challenging problem to treat. Dislocation following RTSA has been reported in the literature to range from 1.5% to 31%.^5^ Patient factors that have been associated with instability events include higher body mass index, mechanical impingement, subscapularis insufficiency, and multiple prior shoulder surgeries. Mechanical factors have been related to multiple implant factors such as glenosphere diameter and humeral socket constraint. Surgical factors include implant position (humeral and/or glenoid version) and iatrogenic neurologic injury.^7^^,^^12^^,^^13^^,^^20^

Treatment of instability following RTSA instability remains a topic of discussion and debate. A trial of closed reduction (CR) is recommended by some as the initial treatment for acute RTSA dislocation without periprosthetic fracture. Chalmers et al,^4^ for example, demonstrated the success of closed treatment in 44% of early (within 3 months) dislocations. This was further supported by Teusink et al^21^ who reported that 62% of patients who were managed with CR of RTSA remained stable >2 years following the instability event.^6^ In contrast, Gerber et al^10^ have indicated that early instability is likely attributable to surgeon error and cannot be managed with CR. Furthermore, CR can hypothetically predispose to a periprosthetic fracture. Although the success rate of CR has be described in multiple studies, there is a paucity of literature discussing polyethylene dissociation in RTSA, nor is there a description of mechanism or risk factors.^17^

The aim of this study is to describe the success of CR for dislocation following RTSA and to report on the mechanism and risk factors for polyethylene dissociation. Our hypothesis is that in certain implant designs there is a risk of polyethylene tray dissociation which will prevent a successful CR.

## Materials and methods

This is a single-center, retrospective review of patients undergoing revision surgery for instability following RTSA.

### Patient selection and data collection

This is an institutional review board–approved study. All revision RTSA cases performed at our institution between September 1, 2013 and March 31, 2021 were reviewed. All cases performed for the diagnosis of prosthetic instability were included in the analysis. Revision surgery cases performed for other causes were excluded.

A total of 22 patients who underwent revision RTSA for instability were identified and included in the current study analysis. Information pertaining to patient’s demographic data, preoperative diagnosis, primary vs. revision RTSA, implant design for the reverse based on humeral tray system (onlay vs. inlay), time to instability event from initial surgery, mechanism of instability, and results of CR were recorded from the retrospective chart review. The presence of polyethylene liner dissociation was determined from the operative notes. Radiographs were analyzed to determine findings of PDH. For analysis purposes, patients were grouped into traumatic and atraumatic (minimal trauma) groups based on etiology and in acute and chronic instability based on time of onset of instability after index RTSA. Although defining acute vs. chronic dislocation is somewhat arbitrary, we used 3 months as has been described in the literature. ^20^^,^^21^

### Treatment of prosthetic instability

Two senior authors (J.D.Z. and Y.W.K.) favored CR as the initial treatment option for dislocation following primary RTSA. One senior author (M.S.V.) does not favor initial CR. For all patients, CR was avoided for delayed presentation and when there was radiographic evidence of polyethylene dissociation.

### Statistical analysis

All statistical analyses were performed using R (R Foundation for Statistical Computing, Vienna, Austria). Wilcoxon rank-sum tests were used to evaluate relationship between continuous and categorical variables. Pearson chi-square analyses were used to evaluate relationship between categorical variables. For all analyses, *P* values <.05 were considered statistically significant.

## Results

### Patient demographics

In total, 22 patients met the inclusion criteria and were included in the analysis. Of the 22 patients with instability 16 patients had primary implants, while 6 patients had instability after a revision surgery. The average time to the initial instability episode from the surgery was 10 months (range 0-44). Fourteen of the 22 patients were male, and the average age of the cohort was 68.8 (range 51-83) (Table I). Five patients had RTSAs with inlay humeral systems (DJO Global Inc., Vista, CA, USA; SMR, Lima Corporate, Arlington, TX, USA) and 17 patients had RTSAs with onlay humeral system (Exactech Inc., Gainesville, FL, USA).

**Table I:** Demographic cohort data.

Characteristic	N = 22∗
Age	
Median (IQR)	69 (64-75)
Mean (SD)	69 (9)
Time from surgery to instability (mo)	
Median (IQR)	4 (1-13)
Mean (SD)	10 (15)
Gender	
Female	8 (36%)
Male	14 (64%)
Body mass index (kg/m^2^)	
Median (IQR)	31.4 (30.3-34.5)
Mean (SD)	31.1 (4.5)
Laterality	
Left	11 (50%)
Right	11 (50%)

*IQR*, interquartile range; *SD*, standard deviation.

∗ n (%).

Thirteen dislocations (59%) occurred acutely (within 3 months of last surgery), and 9 dislocations (41%) occurred later than 3 months. Six patients (27%) had traumatic etiology and 16 patients had atraumatic instability (73%). Of the patients who sustained traumatic dislocations, only 2 of the 6 occurred in the acute postoperative period. Of the patients with atraumatic instability, 11 occurred acutely and 5 occurred after 3 months.

### Treatment of reverse total shoulder arthroplasty instability

**Figure 1:**
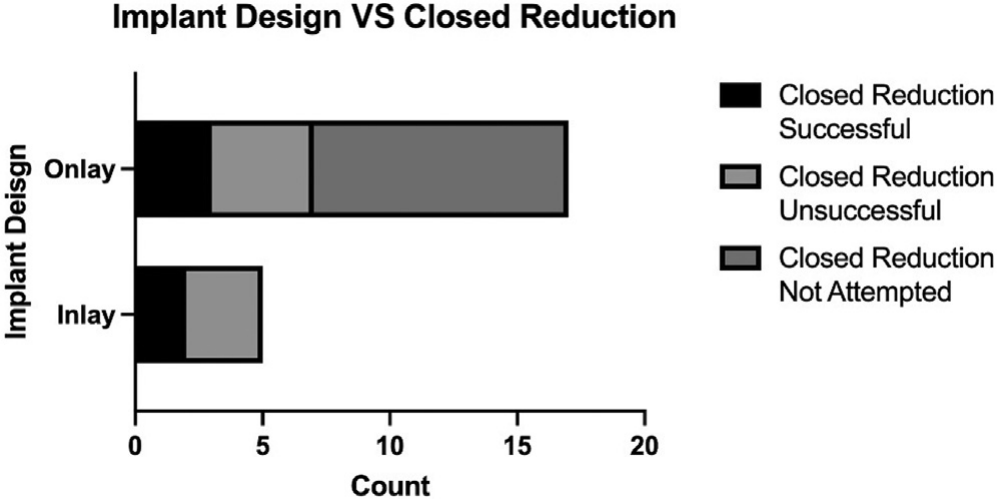
Bar plot demonstrating humeral implant design and variable success of closed reduction after RTSA instability. *RTSA,* reverse total shoulder arthroplasty.

Twelve patients had an initial CR attempted based on attending surgeon’s preference and 5 were successful. There was no statistically significant difference in the success of CR in patients with onlay vs. inlay humeral components (Fig. 1).

All patients in the cohort underwent revision surgery for either initial or recurrent instability, which included revision to RTSA (20) and hemiarthroplasty (2 loose and nonreconstructable base plate [1]; infection [1]). Revision of one or more modular components was performed in patients who retained the RTSA, and this included a combination of revision of glenosphere (7 upsized and/or lateralized), humeral tray (11 upsized), and polyethylene liner revised (13 upsized, thicker liner and/or constrained liner).

**Figure 2:**
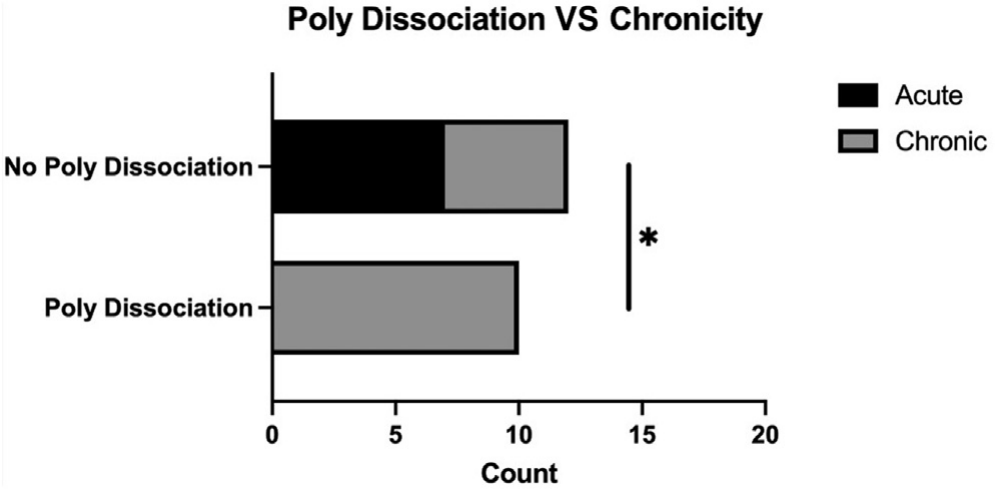
Bar plot demonstrating statistically significant difference in dislocation chronicity and PDH. *PDH,* polyethylene dissociation from humeral tray *∗P<*.05.

**Figure 3:**
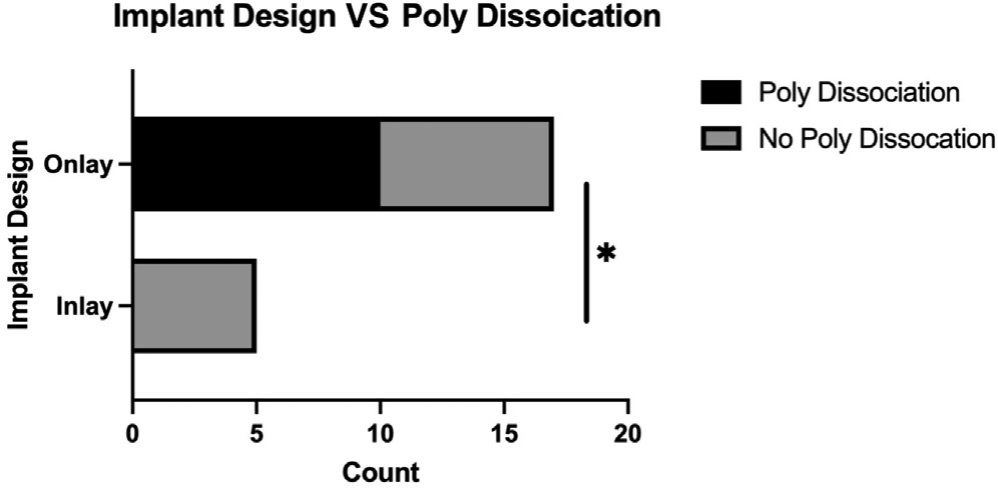
Bar plot demonstrating statistically significant difference in PDH between variable implant design. *PDH,* polyethylene dissociation from humeral tray.

Intraoperatively, it was confirmed that 10 of 22 patients had polyethylene liner dissociation from the humeral tray (PDH). Four of these patients had radiographic evidence of PDH preoperatively, which included findings of apposition of metallic humeral tray and glenosphere and/or presence of soft tissue shadow of dissociated polyethylene liner. All the patients with PDH had late instability (>3 months), which was statistically significant when compared to early dislocations (*P* = .0053; Fig. 2). Regarding humeral component design, all patients in this series with PDH had onlay humeral stems. This association was statistically significant when compared to inlay humeral design (*P* = .0396; Fig. 3).

The mean follow-up after revision RTSA was 23 months (range 1-133) and 7 patients required additional revision procedures. Two patients developed prosthetic joint infection requiring 2-stage revision. One patient sustained a distal humerus fracture after a fall which was treated with open reduction and internal fixation. Four patients had recurrent instability: 1 was a traumatic recurrence (treated with revision surgery) and 3 had atraumatic recurrence (1 refused surgery and the other 2 required additional revision surgeries).

## Discussion

**Figure 4:**
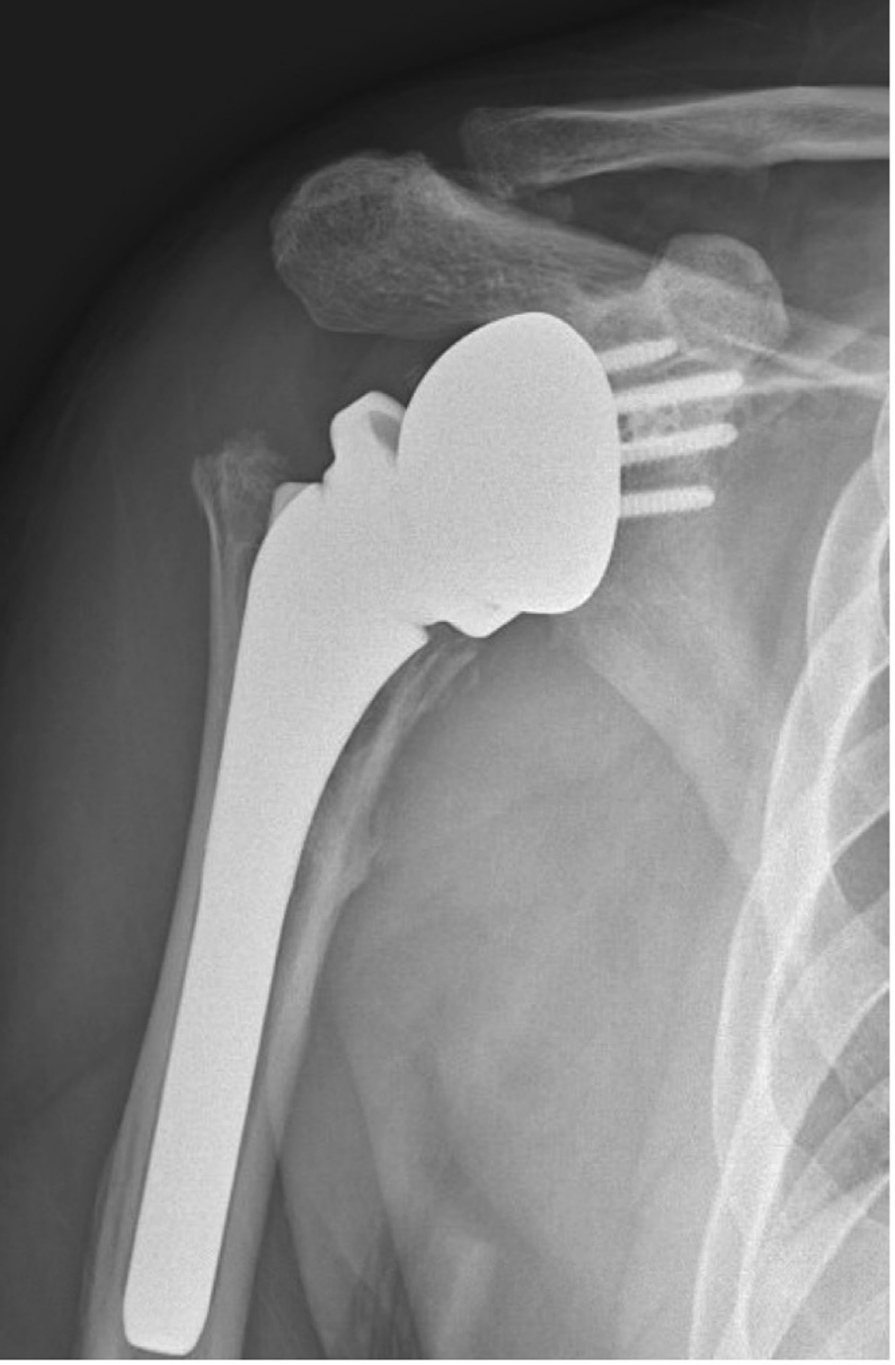
AP radiograph of the right shoulder demonstrating PDH, which should be considered a contraindication to closed reduction of RTSA. *AP*, anteroposterior; *PDH,* polyethylene dissociation from humeral tray; *RTSA,* reverse total shoulder arthroplasty *∗P<*.05.

The results of this study demonstrate that while CR is a possible initial treatment option for RTSA instability, the presence of dissociated polyethylene from humeral tray is an impediment to successful CR. Radiographic workup can help identify PDH, which should be considered a contraindication for CR of RTSA (Fig. 4).

There continues to be debate regarding whether CR should be performed as initial treatment for dislocation following RTSA.^5^^,^^6^^,^^21^ Prior studies have demonstrated that CR can be a successful long-term treatment option for RTSA instability. Perhaps the most robust evidence is from Teusink et al who demonstrated that nearly two-thirds of patients were managed successfully 2 years out from CR.^21^ In this series, patients were selected based on the need for revision surgery and the senior authors viewed CR as initial step prior to open surgery or revision surgery and less commonly as a definitive treatment of dislocated RTSA. The findings from this study are important for surgeons who utilize CR either as initial or potentially definitive treatment of RTSA instability, as the risk of PDH is a definite impediment for CR. In this cohort, having an onlay humeral design was a significant risk factor for PDH, more specifically in patients who present with late instability. Analysis of intraoperative findings from the revision surgeries demonstrated that PDH was present in 10 shoulders. Two variations in PDH were seen; first one in which polyethylene was dissociated from the humeral tray placing the humeral tray in direct contact with glenosphere and the second type in which dissociated polyethylene was still trapped between the humerus and glenosphere. Consequently, the classic radiographic finding of the humeral tray abutted against the metal glenosphere was identified only in the former cases. Therefore, in this scenario we recommend scrutiny of pre- and post-reduction radiographs after attempted CR.

Six of the 10 patients with PDH had an attempted CR but all failed. Retrospective analysis of all 6 radiographs demonstrated radiographic finding of humeral tray in direct contact with the glenosphere. However, this radiographic finding was not present in all cases. It may be possible that those patients who did not have radiographic evidence prior to CR attempt may have had dissociation with the reduction maneuver. Additionally, although this study did not review all patients treated successfully with CR, it is a theoretic possibility that a patient may have had a dissociated tray that could have been reduced or re-engaged with the humeral component during CR, while we feel this is unlikely, the possibility cannot be excluded by this study. This may indicate that the polyethylene was incompletely dissociated as most reduction attempts were performed in office without aggressive maneuvers or sedation. Although we did not have other imaging studies done prior to CR, advanced imaging studies in the form of computed tomography scan or ultrasound may potentially demonstrate a dissociated polyethylene thereby indicating the necessity of open reduction. All patients with PDH in this series had an onlay humeral tray system. Based on findings from this study, onlay humeral tray design may be considered as a risk factor for PDH but more studies with onlay and inlay humeral system RTSA are necessary to validate this finding. Based on the intraoperative and preoperative radiographic findings of PDH, we recommend that PDH should be considered a contraindication for CR of a dislocated RTSA.

Prosthetic instability of RTSA is a difficult problem to treat. The etiology of atraumatic dislocation is poorly understood, and treatment options are associated with high recurrence and revision surgeries. Overall, our revision rate for instability was comparable to that described in the literature with 1.8% of total shoulders done in the collection period requiring revision due to instability. Current literature demonstrates an instability rate of 2.3%-31%. Although our rate is on the lower end of the range, this is likely due to the retrospective nature of this study and is an underrepresentation of the problem.

Additionally, our data set demonstrates that instability is both an early and late problem. The majority of our patients had early dislocations (59%), once again, consistent with current literature. The unique aspect of our subcohort of patients who had PDH is that they all had instability outside of the first 90 days. This leads to the possibility that prolonged poly-wear may impact the locking mechanism of onlay systems predisposing to the dissociation and instability in association with traumatic events.

Although PDH has been previously described in a small case series, our study further expands on this phenomenon. Paynter et al^17^ describe 4 cases of PDH, similarly to our cohort all were in an onlay humeral system. Our study differs from prior literature in the size of our cohort, and the comparison to inlay humeral design. Additionally, the classic radiographic finding of the humeral tray in direct contact with the glenosphere was not present in all our cases of PDH. We suspect that this could be due to many factors—one being a combination of primary polyethylene dissociation resulting in prosthetic instability vs. an instability event resulting in dislocation and simultaneous failure of press-fit mechanism at the polyethylene and humeral tray. We recommend that the surgeon have a high suspicion for PDH and proceed with advanced imaging or additional radiographic views prior to an attempt at CR. A limitation of this study is that it is retrospective in nature. It is very likely possible that all patients with this complication were not identified through a retrospective search. Additionally, the sample size of the study is small but is comparable in size to similar cohorts reported in the literature.

## Conclusion

Dissociation of the polyethylene liner from the humeral tray can be present in an RTSA dislocation and is a contraindication for CR. Although standard radiographs may show the classic finding of the metallic humeral tray articulating directly with the glenosphere, this radiographic sign is only present when the polyethylene liner is completely dislocated.

## Disclaimers

Funding: No funding was disclosed by the authors.

Conflicts of interest: Young W. Kwon is a consultant for DJO Inc. Joseph D. Zuckerman is a consultant for Exactech Inc. Mandeep S. Virk is a consultant for Exactech Inc. The other authors, their immediate families, and any research foundation with which they are affiliated have not received any financial payments or other benefits from any commercial entity related to the subject of this article.
